# Association between Serum Lipid Levels in Greek Children with Dyslipidemia and Mediterranean Diet Adherence, Dietary Habits, Lifestyle and Family Socioeconomic Factors

**DOI:** 10.3390/nu12061600

**Published:** 2020-05-29

**Authors:** Maria Lampropoulou, Maria Chaini, Nikolaos Rigopoulos, Athanasios Evangeliou, Kyriaki Papadopoulou-Legbelou, Antonios E. Koutelidakis

**Affiliations:** 1 Department of Food Science and Nutrition, University of the Aegean, Mitropoliti Ioakim 2, 81440 Myrina, Lemnos, Greece; marylambrop@hotmail.com (M.L.); fns12114@fns.aegean.gr (M.C.); nrigopoulos@aegean.gr (N.R.); 2 4th Department of Pediatrics, Aristotle University of Thessaloniki, “Papageorgiou” General Hospital, 54453 Thessaloniki, Greece; aeevange@auth.gr (A.E.); kpapadopoulou@auth.gr (K.P.-L.)

**Keywords:** dyslipidemia, socioeconomic factors, dietary habits, Mediterranean diet, children

## Abstract

*Background*: Childhood dyslipidemia is an important risk factor for developing cardiovascular disease in adulthood. Our study aimed to investigate a possible correlation between nutritional, lifestyle, behavioral and socioeconomic factors and serum lipid levels in children with dyslipidemia. *Methods*: In this retrospective, observational study, in 31 children with dyslipidemia, aged 3–14 years, dietary habits, physical activity, hours watching television or playing video games, family’s socioeconomic status, weight of children and parents, and duration of breastfeeding were recorded. The children’s adherence to the Mediterranean diet was also evaluated by KidMed index. Statistical analysis was performed using SPSS.22. *Results*: Children with increased physical activity had lower triglyceride levels, compared to those with lower physical activity (*p* = 0.001). Children who consumed only one meal per day, had increased levels of total cholesterol (*p* = 0.01), LDL-cholesterol (*p* = 0.01), ApoB (*p* = 0.001) and lipoprotein (a) (*p* = 0.018), compared to those who consumed more than 3 meals per day (*p* < 0.05). Children who were breastfed less than 6 months had significantly increased LDL-C levels (*p* = 0.022), compared to children who were breastfed more than 6 months. All other parameters investigated did not differ significantly. *Conclusions*: This study showed association between lipid profile of children with dyslipidemia and specific nutritional and socioeconomic factors, such as increased physical activity, increased meals consumption during the day, and exclusive breastfeeding for more than 6 months. Nevertheless, further research is needed, in order to confirm these findings.

## 1. Introduction

Dyslipidemias are disorders characterized by abnormal amounts of blood lipids and lipoproteins [1,2], which are considered to be one of the most important modifiable risk factors for cardiovascular diseases [3,4]. The prevalence of dyslipidemias in childhood shows a large increase in recent years worldwide and increases further when various factors coexist, such as obesity, unhealthy diet and reduced physical activity of children [5,6,7]. Screening for childhood dyslipidemia usually includes measurement of serum levels of total cholesterol (TC), triglycerides (TG), HDL-cholesterol (HDL-C) and LDL-cholesterol (LDL-C) [1,7]. Lipid and lipoprotein level disorders are related to genetic and environmental or socioeconomic factors [2,3]. Dyslipidemias in children, as well as in adults, are divided into primary (caused by genetic factors), and secondary (correlated with dietary habits, drug administration, or the presence of various diseases, such as hypothyroidism, liver and kidney diseases, obesity, etc.) [1,2]. In children, the most common causes of dyslipidemia are genetic factors (particularly familial hypercholesterolemia, FH) [7], which if left untreated, leads to coronary heart disease [8]. The criteria for diagnosis of FH in children include LDL-C levels ≥ 130 mg/dL and family history of FH or premature coronary artery disease (CAD) [9]. 

Diagnosis, management and treatment of dyslipidemias in childhood is of high importance, in order to prevent early atherosclerosis and reduce the incidence of CAD, a leading cause of mortality in adulthood [7]. Several socioeconomic factors have been associated with disorders of lipid levels in children, such as increased screen time [10], reduced physical activity [11], parents’ lower socioeconomic status [12], shorter duration of breastfeeding [13], shorter sleep duration and unbalanced diet [11,14]. The primary treatment of dyslipidemia in children and adolescents include lifestyle modifications, improved dietary habits, and increased physical activity [4,9,15]. The increase of physical activity was correlated with decreased body fat and BMI, higher HDL-C levels, lower TC, TG and LDL-C levels, decreased insulin resistance and lower blood pressure. Therefore, daily moderate or vigorous physical activity and reduced sedentary lifestyle (watching television or playing video games < 2 h/day) should be encouraged in children and adolescents [4]. The important role of nutrition in the management of pediatric dyslipidemia is highlighted in several studies. A beneficial diet includes reduced consumption of total fat and saturated fat, reduced cholesterol and simple sugars consumption and increased intake of complex carbohydrates, without reducing protein intake [5,15]. Specific diets, called CHILD-1 and CHILD-2, have been proposed by the American Academy of Pediatrics, in order to improve children’s lipid profile [3]. In addition, specific children’s dietary habits, such as low consumption of sugar-sweetened beverages, saturated fat, and high calorie products have correlated with decreased BMI, and risk of obesity and metabolic diseases [11,16].

The Mediterranean diet has also been recognized as a possible protective factor in the prevention of CAD. This diet is characterized by high consumption of vegetables, fruits, olive oil, cereals, fishes and low consumption of meat and saturated fats [17,18,19]. Several studies have concluded that specific functional foods of the Mediterranean diet may have a protective effect against atherosclerotic process and thus CAD development. Specific functional foods of the Mediterranean diet such as olive oil, honey, vegetables (tomatoes, cauliflower, broccoli), fruits (citrus fruits, pomegranate, grapes), wild greens (fennel, radish), and herbs (oregano, mint, dittany, salvia) may contribute to CAD prevention; due to the bioactive compounds of those foods, as phytochemicals and polyphenols, such as oleuropain, resveratrol, sulforaphan, anthocyanins, quercetin, tannins etc. Possible mechanisms of their action include improving lipid profile, endothelial factors, thrombotic factors etc. [20,21]. Recent studies concluded that the adherence to the Mediterranean diet may be an important health-promoting dietary approach for children and adolescents and it is affected by socioeconomic factors, parental factors, and modern lifestyle [22]. In children, several studies showed that higher compliance with the Mediterranean diet was associated with higher physical activity, lower body-mass index and psychological distress, better blood pressure regulation, fewer subclinical atherosclerosis markers, and improved metabolic biomarkers [22,23,24]. Giving the fact that children’s compliance with the Mediterranean diet is low [22], improving children’s dietary habits in combination with a healthy lifestyle is of high importance, especially when dyslipidemia coexists. Understanding of various and multifactorial parameters of lifestyle and family influence eating habits is important for the management of children’s dyslipidemia. 

The aim of this study was to investigate the possible association between the Mediterranean diet adherence, nutritional attitudes, lifestyle, behavioral and family socioeconomic factors with serum lipid levels in children and adolescents with dyslipidemia.

## 2. Materials and Methods 

### 2.1. Subjects

The study was performed in accordance with the Helsinki Declaration in 1975, revised in 2013 and the protocol was approved by the Ethic Committee of ‘Papageorgiou’ General Hospital, Thessaloniki, Greece. All the parents of the children, who participated in the study, were informed about the primary target of the study, the confidentiality of data and the voluntary participation and then they signed an informed consent form.

Thirty-one children (18 boys and 13 girls) with familial hypercholesterolemia diagnosed with dyslipidemia, aged 3–14 years, were randomly recruited from the Pediatric Cardiology outpatient unit, of the 4th department of Pediatrics of the Aristotle University of Thessaloniki, at “Papageorgiou” General Hospital. The inclusion criteria were disorders on lipid levels, according to the criteria for dyslipidemia for children [25]. The exclusion criteria were the presence of other disease that causes secondary dyslipidemia (liver, kidney, or thyroid disease etc.) and the administration of any medication.

### 2.2. Study Design

The study was a retrospective, observational study that was conducted from 30-3-2017 to 30-9-2018. During the study, children and their parents, with the assistance of the nutritionists, completed two questionnaires, one for assessing their nutritional, behavioral and social-economic patterns and one for assessing the quality of their diet (adherence to the Mediterranean diet). Parents have been asked to recall their children’s nutritional and behavioral habits over the past year.

### 2.3. Questionnaires

The first questionnaire was based on the PANACEA study validated questionnaire with some modifications [26,27] and was evaluated socio-demographic, nutritional and lifestyle characteristics of children. The questionnaire included two parts. First part: questions about gender, age, weight and height of both children and parents, parental employment, family income and education of the parents, birth weight of children, hours watching television or video games playing, physical activity into and out of the school. Second part: questions concerning nutritional habits of children and especially daily meals, breakfast consumption, frequency of basic food groups consumption. The possible answers were “every day”, “1–2 times a week”, “3–4 times a week”, “5–6 times a week” and “never.

In the second questionnaire the Mediterranean Diet Index (KIDMED Index) was measured. This quality index was designed for children and it is based on the Mediterranean Diet characteristics [28]. The questionnaire was consisted of 15 questions about the dietary habits, in order to detect the quality of diet and especially the adherence to the Mediterranean diet. The possible answers were “yes” (codified as 1) or “no” (codified as 0). KIDMED Index > 7 indicates the highest adherence to the Mediterranean diet, 4–7 moderate and < 4 low adherence.

### 2.4. Biochemical Analyses

The lipid profile {TC, LDL-C, HDL-C, TG, apolipoprotein A_1_ (ApoA1), apolipoprotein B (ApoB) and lipoprotein a (Lpa) levels} was assessed in a morning blood sample after a 12-hour fast, using an Architect c16000 Automatic Biochemistry Analyzer (Abbott, U.S., Chicago, Ilinois, Abbott, 100 Abbott Park Road, Abbott Park, Ill. 60064). 

### 2.5. Statistical Analysis

The statistical analysis was performed using Statistical Package for Social Sciences (SPSS) ver. 22 (IBM, 1New Orchard Road Armonk, New York 10504–1722United States). Kolmogorov-Smirnov test was used in order to justify the normality of distribution. The test resulted in a normal distribution of data for all lipid parameters. In order to investigate the possible correlation between blood lipid levels and lifestyle, nutritional and socio-demographic characteristics one-way analysis of variance (one-way ANOVA, using Bonferoni test) and *t*-test were used. One-way analysis was used for the correlation of all nutritional parameters, extracurricular activities, as well as the weight of children and parents, with lipid levels, while *t*-test was used for the correlation of all the behavioral, lifestyle and socioeconomic factors with lipid levels. The results expressed as mean ± SD in all situations. Differences were considered significant at *p* < 0.05, coefficient interval 95%.

An online statistical sample size calculator was used for the calculation of sample size. In order to achieve a power of 80% and a level of significance of 5%, for detecting a true difference in total cholesterol and LDL- cholesterol levels about 15%, the study required a sample size of 22 children (31 children finally included).

## 3. Results

The majority of children who participated in the study were aged between 5 and 12 years, had normal weight, moderate adherence to the Mediterranean diet, increased hours of watching television/playing video games and moderate-high physical activity (All demographic and behavioral characteristics of the participants are summarized Table 1).

The comparison between children’s serum lipid levels and behavioral and socioeconomic characteristics showed no correlation between serum lipid levels and hours of watching television or playing video games, children’s weight classification and children’s weight at birth (*p* > 0.05). Children with increased hours of sports activities had lower triglyceride levels, compared to children with fewer hours of sports activity (*p* = 0.001). The increased hours of extracurricular activities were associated with decreased levels of ApoB (*p* = 0.023). Children who were breastfed less than 6 months had significantly increased LDL-C levels (*p* = 0.022) compared to children who were breastfed more than 6 months (Table 2).

There was no association between children’s serum lipid levels and parental income, educational level, employment and BMI (Table 3). Furthermore, there was no significant association between children’s serum lipid levels and Mediterranean diet compliance, breakfast consumption, consumption of fruits, vegetables, fresh juices, deserts and sodas (*p* > 0.05). Children who ate only one meal per day, showed increased levels of total cholesterol, LDL-C, Apo B and Lp(a), compared to those who consumed 2, 3 or more than 3 meals per day (*p* = 0.01, 0.01, 0.001, and 0.018 respectively) (Table 4). 

## 4. Discussion

The last decades various factors such as lifestyle, dietary, social, economic, and parental factors have been studied as possible parameters that affect children’s lipid and glucose levels. They have also been associated with obesity, diabetes mellitus and children’s overall health [12,16,29,30]. These factors are of high importance, especially in children with elevated serum lipid levels [9]. 

The present study investigated the possible association between nutritional, lifestyle, behavioral and socioeconomic factors with lipid profile in children diagnosed with dyslipidemia. A main finding of the study was that children who consumed only one meal per day, had increased levels of total cholesterol, LDL-C, ApoB and Lp(a), compared to children who consumed two, three or more than three meals per day. These data are in accordance with many previous studies in the literature, which have shown that the increased number of meals per day was associated with improved biochemical indicators and reduced risk of childhood obesity [16,31,32,33,34,35]. More specifically, skipping meals, especially breakfast, has been associated with decreased nutrients intake and increased body weight in children and adolescents [16,31,32,33]. Furthermore, it has been associated with cardiometabolic risk factors in adulthood, such as insulin resistance and elevated LDL-C levels [34]. The above findings could be related to a possible postprandial hyperglycemia and lipemia [36]. Data from the cross-sectional Hellenic National Nutrition and Health Survey in 598 children and adolescents aged 4– 19 years, showed that increased snack consumption was positively correlated with vitamin D, vitamin K, riboflavin, niacin, pantothenic acid, folate, magnesium, copper and selenium intake [37]. A metanalysis of 47 studies who investigated the correlation between breakfast consumption and nutritional adequacy, body weight and academic performance in children and adolescents showed that the consumption of a healthy breakfast consisting of a variety of foods on a daily basis (particularly whole grains, rich with roughage and nutrients, fruit and dairy products) is beneficial for children’s health [31]. In a large epidemiological study conducted in a total of 7.055 obese children, who lived in the area of Incheon, showed that elevated TG and LDL-C levels were positively correlated with food overconsumption, unbalanced meals and omission of breakfast [35]. In other studies, a lifestyle pattern characterized by high frequency eating, breakfast consumption and higher adherence to the Mediterranean diet, was negatively correlated with child’s BMI [16], while the consumption of ultra-processed products was a predictor for increased total and LDL cholesterol levels in preschoolers [38]. Nevertheless, in the present study no correlation was observed between lipid levels and breakfast habits or food groups consumption.

Another important finding of the present study was that although most children reported moderate (64.5%) or high (22.6%) compliance with the Mediterranean diet (MD), KidMed index was not correlated with children’s lipidemic profile. KidMed index is a useful tool that investigates the degree of children’s compliance with the standard Mediterranean Diet; the increased KidMedIndex score has been correlated with increased nutrients intake in children and adolescents [28,39]. Many studies, using KidMed Index, have observed that the children’s adherence to the Mediterranean diet is low in most countries [22,40]. A study from Spain with 1.177 children and adolescents, observed that 59% of participants had suboptimal adherence to the Mediterranean Diet [41], while another study from Cyprus with 1.140 children, showed that 37% of children had a poor KIDMED score [42]. In a study of 359 students from Greece, the percentage of adherence to the Mediterranean Diet was positively correlated with improved health-related quality of life in adolescents [43]. Other studies have also shown that the adherence to the Mediterranean Diet, was associated with improved arterial stiffness and fewer risk factors for metabolic syndrome and obesity in children [16,44,45]. Thus, the basic strategy is to improve dietary habits based on adherence to the Mediterranean diet, especially in children with dyslipidemia. 

In our study it was also found that children who exercised once a week had higher triglyceride levels, compared with those who participated in sports activities more than twice a week. Our findings are in accordance with another Greek study in 2.043 school-age children (9–13 years), in which it was observed that a lifestyle pattern with moderate to vigorous levels of physical activity was negatively correlated with total cholesterol, LDL-C and total/HDL-C levels [11]. In another study of 1.235 adolescents aged 12–19 years, the odds ratio for high-risk HDL-C and triglyceride values decreased linearly with increasing minutes of moderate-to-vigorous physical activity [46]. Nevertheless, our study found no association between serum lipid levels and hours of watching television or playing video games. Other studies, showed that a lifestyle pattern of more screen time, was inversely associated with HDL cholesterol levels [11], while a recent study in 600 children, showed that every extra hour of watching television was associated with a 7.0% increase in triglyceride levels and 2.6% decrease in HDL-C levels [10]. In addition, a study in U.S.A. in 4.069 children found a lower incidence of obesity in children who watched fewer hours of television per day, compared to those who watched more hours per day [47]. A possible explanation is that the sedentary lifestyle, while watching TV, is correlated with reduced energy expenditure, lower basic metabolism and increased consumption of high calorie products, rich in sugar and fat, such as sodas, chips, cakes, sweets, etc. [16].

Another important finding of our study was that children who were breastfed less than 6 months had statistically significantly increased LDL-C levels compared to children who were breastfed more than 6 months. This is in accordance with a recent study in Hong Kong, where 3.261 exclusively breastfed children had lower total cholesterol and LDL-C levels, compared with formula feeding children [13]. In another study, children who were breastfed, had significantly lower systolic blood pressure and TG levels, and higher HDL-C levels, compared to children who have never breastfed [48]. Breastfeeding is an important process, which is influenced by biological factors, habits, patterns and behaviors that exist in every society. It seems to have a protective effect against hypertension, metabolic syndrome, and diabetes, while data on postpartum weight and lipid profile are controversial [49].

Social and economic factors that affect family life, play an important role for children’s dietary habits and their overall health and cardiovascular disease progression, especially in children with dyslipidemia. In the present study, the higher parents’ education level was not correlated with children’s improved lipids levels. In a Turkish study in 1.044 children, aged 12 and 13 years, authors observed that the educational level of the parents had a positive correlation with TC and HDL-C levels and a negative correlation with TC/HDL ratio for both boys and girls. The study concluded that the level of education of both parents, is crucial for the development of specific health behaviors [50]. Another study in Greece showed that mothers with higher education level, had healthier dietary choices for their children, with the potential effect of improving lipid profile [51]. In addition, no association was found in the present study between parents’ economic status and children’s lipid profile. Nevertheless, other studies have shown controversial results. A review study reports negative correlation between family socioeconomic status and childhood obesity [30]. In another study, children with lower socioeconomic status of the family had higher triglyceride and glucose levels and increased risk of metabolic syndrome and diabetes in adulthood [12]. So, parents play an important role in guiding their children to healthier eating habits and more physical activity [52]. 

With the rise in childhood obesity, many studies have focused on family and social influences on their children’s dietary attitudes. Family culture and lifestyle may affect children’s nutritional choices, which tend to consume more foods that are readily available and cooked easily and quickly. Other important factors that affect the amount of food consumed and children’s eating habits are whether families eat together, watching television during the meal and the source of food (homemade or not), which can also affect their lipid profile [53]. A study in Greek adolescents living in rural areas, observed that those who ate from the school canteen or in fast food restaurants, had higher consumption of ‘junk food’ [51]. In a cross-sectional study of 402 children aged 4–7 years a statistically significant correlation was observed between family history of dyslipidemia and total cholesterol, LDL-C and TG levels. It has also been found a correlation between early weaning and LDL-C levels, between sedentarism and LDL-C and TG levels, as well as between HDL-C levels and candy consumption [16,54].

Many epidemiological studies in recent years highlighted the important role of nutritional habits, lifestyle, behavioral and socioeconomic factors of families in the overall health of children and adolescents and in reducing the incidence of chronic diseases (such as obesity, diabetes, cardiovascular diseases, hypertension and metabolic syndrome). To our knowledge, few studies have been correlated the above factors with dyslipidemia in childhood. In dyslipidemic children, either of genetic or other etiology, the risk of chronic disease progression is increased in childhood and adulthood. Therefore, lifestyle changes and the avoidance of predisposing factors are important for the management of dyslipidemia [5,9]. The present retrospective study may provide primary data on the possible effect of these factors on children with dyslipidemia; nevertheless, larger epidemiological studies are needed, in order to ensure and extent these results. Although in our study there was a correlation between lipid levels and physical activity, increased number of meals per day and breastfeeding, no correlation was observed between serum lipid levels and adherence to the Mediterranean diet, food groups consumption, screen time, children’s weight and weight at birth, as well as parental BMI, income, employment and educational level.

The present study has some limitations. Initially, there is revocation error and selection error. Accurate and detailed data were gathered to determine the revocation error. Furthermore, this is a retrospective study and thus the results should be further confirmed by large prospective studies. The study sample is limited, especially in specific subgroups, and includes children with dyslipidemia that were visited only one hospital in Northern Greece. Larger epidemiological studies are needed in children from all parts of the country (urban, rural and island regions), in order to extract more reliable conclusions regarding the effect of socioeconomic factors in children with dyslipidemia.

## 5. Conclusions 

This study showed a correlation between lipid profile of children with dyslipidemia and specific nutritional, lifestyle, behavioral and socioeconomic factors. Our results could be used to design nutritional policies and promote healthy nutritional habits, adherence to the Mediterranean diet, and the adoption of a healthier lifestyle, in order to improve children’s lipid profile. 

## Figures and Tables

**Table 1:** General socio-demographic and behavioral characteristics of children and their parents.

Sample Characteristics		Number (%)
**Gender**	Boys Girls	18 (58.1) 13 (41.9)
**Age**	Up to 5 5 to 12 12 and above	2 (6.5) 25 (80.6) 4 (12.9)
**Hours watching television and playing video games**	1–2 h/week 3–4 h/week 5–6 h/week Daily	0 (0.0) 2 (6.5) 4 (12.9) 25 (80.6)
**Extracurricular Activities**	1 time/week 2 times/week > 2/week	6 (19.4) 12 (38.7) 13 (41.9)
**Sports Activities**	1 time/week 2 times/week > 2/week	10 (32.3) 2 (6.5) 19 (61.3)
**Birth Weight**	< 2,5 kg 2,5 –4,0 kg > 4,0 kg	7 (22.6) 24 (77.4) 0 (0.0)
**Parent’s Education**	Primary Secondary High	3 (9.7) 14 (45.2) 14 (45.2)
**Parental Employment**	Employed Unemployed	23 (74.2) 8 (25.8)
**Family Income**	< 15,000 euro 15,000 –30,000 euro > 30.000 euro	13 (41.9) 16 (51.6) 2 (6.5)
**Parent’s Body Mass Index (BMI)**	< 18.5 (underweight) 18.5–24.99 (normal) 25–29.99 (overweight) >30 (obese)	1 (3.2) 15 (48.4) 11 (35.5) 4 (12.9)
**Children’s weight evaluation (based on IOTF cut-off points)**	underweight normal weight overweight obese	3 (10.0) 16 (53.3) 6 (20.0) 5 (16.7)
**Mediterranean Diet Quality Index (Kidmed score)**	<4 4–7 >7	4 (12.9) 20 (64.5) 7 (22.6)

Kidmed Score: <4: low adherence, 4–7: moderate adherence, >7: high adherence.

**Table 2:** Association between children’s serum lipid levels and their lifestyle, behavioral and socioeconomic characteristics.

**Watching Television or Playing Video Games (Hours Per Week)**					
**Children’s Serum Lipid Levels**	**3–6 h** **(*n* = 6)**	**Daily** **(*n* = 25)**			***p-v***
Total Cholesterol (mg/dL) Triglycerides (mg/dL) HDL cholesterol (mg/dL) LDL cholesterol (mg/dL) ApoA (mg/dL) ApoB (mg/dL) Lp(a) (mg/dL)	240.8 ± 46.2 87.7 ± 50.5 43.2 ± 8.5 181.3 ± 44.4 131.9 ± 13.1 115.3 ± 21.2 45.1 ± 21.5	209.8 ± 51.2 98.8 ± 76.1 56.3 ± 14.2 134.8 ± 51.0 148.4 ± 29.8 100.5 ± 34.9 61.7 ± 46.2			0.495 0.379 0.205 0.630 0.062 0.315 0.448
**Extracurricular Activities (Times/Week)**					
**Children’s Serum Lipid Levels**	**1** **(*n* = 6)**	**2** **(*n* = 12)**	**> 2** **(*n* = 13)**		***p-v***
Total Cholesterol (mg/dL) Triglycerides (mg/dL) HDL cholesterol (mg/dL) LDL cholesterol (mg/dL) ApoA (mg/dL) ApoB (mg/dL) LpA (mg/dL)	214.3 ± 50.0 118.0 ± 70.5 49.66 ± 12.7 141.0 ± 58.6 137.2 ± 39.0 129.2 ± 32.8a 29.4 ± 25.0	236.2 ± 48.6 95.9 ± 75.1 59.2 ± 14.4 161.0 ± 48.8 146.5 ± 30.0 116.3 ± 36.8a 48.0 ± 33.5	196.6 ± 50.0 87.4 ± 71.8 50.8 ± 13.7 129.2 ± 52.4 145.7 ± 24.12 87.61 ± 20.3b 74.2 ± 48.2		0.154 0.700 0.258 0.326 0.850 0.023 * 0.164
**Sports Activities (Times/Week)**					
**Children’s Serum Lipid Levels**	**1-2** **(*n* = 12)**	**> 2** **(*n* = 19)**			***p-v***
Total Cholesterol (mg/dL) Triglycerides (mg/dL) HDL cholesterol (mg/dL) LDL cholesterol (mg/dL) ApoA (mg/dL) ApoB (mg/dL) LpA (mg/dL)	208.7 ± 57.2 146.7 ± 71.3a 52.7 ± 14 128.6 ± 55 144 ± 26.9 100.9 ± 35.2 60.6 ± 48.6	219.5 ± 48.0 65.0 ± 25.6b 54.2 ± 14.3 153.5 ± 49.9 144.9 ± 28.7 105.6 ± 32.0 57.2 ± 40.5			0.548 0.001 * 0.785 0.916 0.994 0.581 0.730
**Child’s Weight at Birth**					
**Children’s Serum Lipid Levels**	**< 2,5 kg** **(*n* = 7)**	**2,5 – 4,0 kg** **(*n* = 24)**			***p-v***
Total Cholesterol (mg/dL) Triglycerides (mg/dL) HDL cholesterol (mg/dL) LDL cholesterol (mg/dL) ApoA (mg/dL) ApoB (mg/dL) LpA (mg/dL)	221.4 ± 67.5 76.4 ± 32.3 52.0 ± 11.7 157.8 ± 66.4 137.1 ± 26.4 122.4 ± 39.2 53.4 ± 19.9	213.6 ± 46.9 102.5 ± 78.7 54.12 ± 14.7 139.7 ± 48.7 147.4 ± 28.2 97.1 ± 27.9 60.3 ± 49.1			0.729 0.403 0.746 0.434 0.412 0.079 0.722
**Duration of Breastfeeding**					
**Children’s Serum Lipid Levels**	**< 6 months** **(*n* = 10)**	**> 6 months** **(*n* = 21)**			***p-v***
Total Cholesterol (mg/dL) Triglycerides (mg/dL) HDL cholesterol (mg/dL) LDL cholesterol (mg/dL) ApoA (mg/dL) ApoB (mg/dL) LpA (mg/dL)	202.3 ± 33.5 97.4 ± 41.7 51.2 ± 11.9 134.0 ± 30.4a 139.7 ± 24.6 105.5 ± 28.4 58.3 ± 42.7	186.0 ± 58.3 69.0 ± 42.4 44.0 ± 8.12 97.5 ± 36.6b 137.5 ± 17.6 73.5 ± 3.5 84.5 ± 13.4			0.120 0.145 0.542 0.022 * 0.943 0.345 0.554
**Children’s Weight Evaluation (Based on IOTF Cut-off Points)**					
**Children’s Serum Lipid Levels**	**Underweight** **(*n* = 3)**	**Normal** **(*n* = 16)**	**Overweight** **(*n* = 6)**	**Obese** **(*n* = 5)**	***p-v***
Total Cholesterol (mg/dL) Triglycerides (mg/dL) HDL cholesterol (mg/dL) LDL cholesterol (mg/dL) ApoA (mg/dL) ApoB (mg/dL) LpA (mg/dL)	187.0 ± 42.2 122.3 ± 47.0 57.5 ± 20.5 117.6 ± 34.0 134.6 ± 145.9 98.6 ± 30.8 98.9 ± 32.4	212.7 ± 56.9 76.3 ± 48.8 54.5 ± 14.6 144.0 ± 57.1 145.9 ± 25.1 109.8 ± 40.2 67.0 ± 50.0	211.0 ± 41.7 76.8 ± 49.9 57.3 ± 16.4 137.9 ± 47.9 144.4 ± 41.3 95.8 ± 17.3 32.3 ± 25.3	238.4 ± 52.0 129.0 ± 102.6 44.8 ± 7.0 167.6 ± 61.0 147.6 ± 17.8 100.0 ± 27.3 42.5 ± 20.8	0.600 0.280 0.492 0.642 0.932 0.844 0.170

* *pv* < 0.05 (highlighted with bold) represents statistically significant differences among lipid values in different answers. Different letters in each line represents the difference among the answers (one Way Anova, Bonferoni test or *t*-test).

**Table 3:** Association between children’s serum lipid levels and behavioral and socioeconomic characteristics of their parents.

**Parental Education Level**				
**Children’s Serum Lipid Levels**	**Primary or Secondary** **(*n* = 17)**	**Higher** **(*n* = 14)**		***p-v***
Total Cholesterol (mg/dL) Triglycerides (mg/dL) HDL cholesterol (mg/dL) LDL cholesterol (mg/dL) ApoA (mg/dL) ApoB (mg/dL) LpA (mg/dL)	231.3 ± 48.4 96.0 ± 67.3 55.9 ± 14.8 157.3 ± 50.5 149.4 ± 30.4 114.3 ± 38.6 51.3 ± 45.8	196.0 ± 49.1 97.3 ± 78.5 50.7 ± 12.9 127.4 ± 52.0 139.1 ± 24.1 91.9 ± 18.9 69.0 ± 37.1		0.772 0.710 0.834 0.717 0.391 0.081 0.559
**Parental Employment**				
**Children’s Serum Lipid Levels**	**Employed** **(*n* = 23)**	**Unemployed** **(*n* = 8)**		***p-v***
Total Cholesterol (mg/dL) Triglycerides (mg/dL) HDL cholesterol (mg/dL) LDL cholesterol (mg/dL) ApoA (mg/dL) ApoB (mg/dL) LpA (mg/dL)	207.0 ± 51.3 108.3 ± 78.8 52.0 ± 12.4 134.9 ± 51.6 139.7 ± 27.0 97.9 ± 27.8 62.6 ± 45.8	239.2 ± 45.3 63.1 ± 23.6 58.3 ± 17.7 169.3 ± 49.2 155.8 ± 27.2 117.5 ± 40.0 49.5 ± 35.9		0.128 0.125 0.279 0.112 0.173 0.162 0.487
**Family Income (euro)**				
**Children’s serum lipid Levels**	**<15,000** **(*n* = 13)**	**>15,000** **(*n* = 18)**		***p-v***
Total Cholesterol (mg/dL) Triglycerides (mg/dL) HDL cholesterol (mg/dL) LDL cholesterol (mg/dL) ApoA (mg/dL) ApoB (mg/dL) LpA (mg/dL)	228.4 ± 45.4 123.1 ± 88.8 56.0 ± 11.7 150.0 ± 46.7 150.8 ± 23.6 114.3 ± 41.5 53.8 ± 38.4	205.9 ± 54.1 77.5 ± 49.8 52.1 ± 15.5 139.4 ± 57.3 140.2 ± 30.2 96.3 ± 22.6 62.6 ± 47.3		0.519 0.066 0.246 0.452 0.346 0.168 0.506
**Parents’ Weight Evaluation (According BMI, WHO)**				
**Children’s Serum Lipid Levels**	**Underweight/Normal** **(*n* = 16)**	**Overweight** **(*n* = 11)**	**Obese (Class I or II)** **(*n* = 4)**	***p-v***
Total Cholesterol (mg/dL)		193.0 ± 50.6	183.7 ± 23	0.071
Triglycerides (mg/dL)	196.0 ± 0	115.0 ± 57.0	157 ± 130	0.512
HDL cholesterol (mg/dL)	62.0 ± 0	49.8 ± 12.3	56.5 ± 23.3	0.542
LDL cholesterol (mg/dL)	56.0 ± 0	122.1 ± 49.3	101.3 ± 10.7	0.095
ApoA (mg/dL)	128.0 ± 0	136.7 ± 30.4	134.1 ± 19.5	0.634
ApoB (mg/dL)		102.1 ± 34.3	89.0 ± 7.1	0.398
LpA (mg/dL)		59.4 ± 54.1	68.8 ± 32.2	0.782

*pv* < 0.05 represents statistically significant differences among lipid values in different answers. Different letters in each line represents the difference among the answers (one Way ANOVA, Bonferoni test or *t*-test).

**Table 4:** Association between children’s serum lipid levels and their nutritional attitudes.

**Breakfast Consumption (Days/Week)**						
**Children’s Serum Lipid Levels**	**1–3** **(*n* = 5)**	**4–5** **(*n* = 4)**	**6–7** **(*n* = 22)**			***p-v***
Total Cholesterol (mg/dL) Triglycerides (mg/dL) HDL cholesterol (mg/dL) LDL cholesterol (mg/dL) ApoA (mg/dL) ApoB (mg/dL) LpA (mg/dL)	217.0 ± 13.2 64.0 ± 32.0 56.6 ± 18.5 147.8 ± 16.8 165.6 ± 23.0 99.4 ± 20.0 31.4 ± 23.8	205.5 ± 87.6 116.7 ± 71.8 43.7 ± 11.5 140.0 ± 97.1 136.0 ± 18.0 101.6 ± 29.3 89.4 ± 61.1	216.8 ± 50.8 100.4 ± 77.4 54.9 ± 13.1 143.6 ± 50.3 140.3 ± 28.2 105.6 ± 36.7 60.8 ± 41.2			0.923 0.506 0.315 0.977 0.166 0.929 0.164
**Main Meals Consumed During the Day**						
**Children’s Serum Lipid Levels**	**1** **(*n* = 5)**	**2** **(*n* = 6)**	**3** **(*n* = 14)**	**> 3** **(*n* = 6)**		***p-v***
Total Cholesterol (mg/dL) Triglycerides (mg/dL) HDL cholesterol (mg/dL) LDL cholesterol (mg/dL) ApoA (mg/dL) ApoB (mg/dL) LpA (mg/dL)	301.5 ± 7.7a 67.0 ± 4.2 52.5 ± 0.7 244.5 ± 7.7a 129.5 ± 4.9 176.0 ± 0.0a 47.4 ± 14.2a	225.2 ± 54.8b 88.7 ± 67.4 52.8 ± 15.7 154.5 ± 60.9b 157.2 ± 29.1 112.2 ± 31.5b 23.2 ± 20.1b	198.0 ± 40.2b 94.0 ± 63.5 55.6 ± 14.4 126.2 ± 35.0b 144.1 ± 25.8 91.6 ± 20.7b 78.6 ± 42.0a	245.5 ± 43.1b 182.5 ± 176.0 40.5 ± 2.2 168.0 ± 80.6b 120.0 ± 42.4 95.5 ± 34.6b 23.1 ± 0b		0.01 * 0.352 0.561 0.01 * 0.312 0.001 * 0.018 *
**Fruit Consumption (Times/Week)**						
**Children’s Serum Lipid Levels**	**1** ***–*2** **(*n* = 8)**	**3** ***–*4** **(*n* = 7)**	**5** ***–*6** **(*n* = 3)**	**Daily** **(*n* = 10)**	**None** **(*n* = 3)**	***p-v***
Total Cholesterol (mg/dL) Triglycerides (mg/dL) HDL cholesterol (mg/dL) LDL cholesterol (mg/dL) ApoA (mg/dL) ApoB (mg/dL) LpA (mg/dL)	215.1 ± 54.4 98.8 ± 87.8 50.1 ± 16.4 145.8 ± 58.3 149.0 ± 23.3 98.3 ± 22.8 47.1 ± 18.7	247.5 ± 59.0 120.1 ± 90.6 55.6 ± 11.0 171.7 ± 63.7 123.6 ± 28.2 131.6 ± 42.0 49.8 ± 30.8	200.6 ± 40.6 97.6 ± 61.1 52.6 ± 9.0 128.6 ± 39.92 134.7 ± 18.6 103.5 ± 31.9 157.7 ± 0	211.5 ± 43.4 81.8 ± 59.9 59.7 ± 14.9 137.1 ± 46.1 165.5 ± 26.2 103.4 ± 31.9 53.1 ± 57.2	168.6 ± 30.5 84.3 ± 39.1 40.3 ± 3.5 111.3 ± 35.2 129.4 ± 19.7 74.6 ± 9.5 83.4 ± 34.8	0.235 0.880 0.274 0.495 0.061 0.175 0.095
**Vegetable Consumption (Times/Week)**						
**Children’s Serum Lipid Levels**	**1** ***–*2** **(*n* = 6)**	**3–4** **(*n* = 9)**	**5–6** **(*n* = 2)**	**Daily** **(*n* = 11)**	**None** **(*n* = 3)**	***p-v***
Total Cholesterol (mg/dL) Triglycerides (mg/dL) HDL cholesterol (mg/dL) LDL cholesterol (mg/dL) ApoA (mg/dL) ApoB (mg/dL) LpA (mg/dL)	177.5 ± 43.6 105.3 ± 63.6 43.0 ± 7.2 113.5 ± 46.6 143.4 ± 19.3 88.8 ± 25.6 63.3 ± 39.2	206.7 ± 55.4 104.1 ± 78.2 58.2 ± 17.6 132.4 ± 55.6 146.6 ± 32.6 93.3 ± 17.8 65.8 ± 30.8	218.0 ± 21.2 103.5 ± 91.2 54.5 ± 16.2 144.0 ± 18.3 145.4 ± 34.7 105.5 ± 37.4 124.0 ± 0	240.4 ± 52.2 88.0 ± 78.4 52.3 ± 9.3 171.0 ± 56.2 135.5 ± 23.7 123.0 ± 42.8 55.3 ± 55.1	223.3 ± 11.2 83.6 ± 74.8 67.3 ± 18.5 139.0 ± 10.8 180.5 ± 31.8 97.0 ± 11.3 13.3 ± 16.1	0.177 0.981 0.112 0.252 0.371 0.284 0.189
**Fresh Juice Consumption (Times/Week)**						
**Children’s Serum Lipid Levels**	**1–2** **(*n* = 8)**	**3–4** **(*n* = 4)**	**5–6** **(*n* = 2)**	**Daily** **(*n* = 9)**	**None** **(*n* = 8)**	***p-v***
Total Cholesterol (mg/dL) Triglycerides (mg/dL) HDL cholesterol (mg/dL) LDL cholesterol (mg/dL) ApoA (mg/dL) ApoB (mg/dL) LpA (mg/dL)	238.2 ± 59.4 94.0 ± 88.1 51.1 ± 10.8 169.3 ± 61.6 143.0 ± 10.6 112.8 ± 47.7 76.3 ± 44.9	237.7 ± 61.0 74.0 ± 26.7 48.7 ± 13.9 176.0 ± 62.9 137.3 ± 22.1 106.1 ± 19.6 77.5 ± 40.6	195.5 ± 64.3 75.5 ± 70.0 55.0 ± 0 140.0 ± 57.9 114.0 ± 12.7 106.5 ± 19.0 41.8 ± 48.2	193.7 ± 48.2 109.1 ± 90.3 52.3 ± 13.9 119.5 ± 48.0 140.5 ± 36.6 94.3 ± 15.0 53.3 ± 31.3	210.6 ± 34.8 101.8 ± 55.2 60.1 ± 17.9 130.6 ± 30.0 162.8 ± 30.3 101.4 ± 38.6 44.7 ± 53.0	0.378 0.935 0.666 0.212 0.203 0.909 0.628
**Dessert Consumption (Times/Week)**						
**Children’s Serum Lipid Levels**	**1–2** **(*n* = 12)**	**3–4** **(*n* = 9)**	**5–6** **(*n* = 3)**	**Daily** **(*n* = 6)**	**None** **(*n* = 1)**	***p-v***
Total Cholesterol (mg/dL) Triglycerides (mg/dL) HDL cholesterol (mg/dL) LDL cholesterol (mg/dL) ApoA (mg/dL) ApoB (mg/dL) LpA (mg/dL)	221.3 ± 56.4 98.6 ± 73.2 48.0 ± 10.8 156.5 ± 51.5 131.9 ± 21.3 109.4 ± 31.4 55.8 ± 28.8	233.5 ± 57.0 104.3 ± 86.1 53.1 ± 16.8 160.1 ± 63.4 142.6 ± 33.7 111.6 ± 43.0 56.5 ± 46.8	215.6 ± 15.5 101.6 ± 65.6 61.6 ± 4.5 134.0 ± 2.6 170.5 ± 0.7 89.0 ± 14.1 46.7 ± 67.0	186.1 ± 30.7 85.1 ± 67.3 59.8 ± 16.7 109.1 ± 34.3 158.7 ± 24.2 93.3 ± 11.8 51.6 ± 31.8	155.0 ± 0 57.0 ± 0 61.0 ± 0 83.0 ± 0 156.0 ± 0 69.0 ± 0 159.7 ± 0	0.338 0.970 0.396 0.230 0.278 0.613 0.195
**Sodas Consumption (Times/Week)**						
**Children’s Serum Lipid Levels**	**1–2** **(*n* = 4)**	**3–4** **(*n* = 1)**	**None** **(*n* = 26)**			***p-v***
Total Cholesterol (mg/dL) Triglycerides (mg/dL) HDL cholesterol (mg/dL) LDL cholesterol (mg/dL) ApoA (mg/dL) ApoB (mg/dL) LpA (mg/dL)	239.5 ± 32.3 110.7 ± 127.0 56.7 ± 22.0 160.5 ± 43.7 148.0 ± 56.5 94.6 ± 23.0 42.8 ± 39.1	211.0 ± 0 38.0 ± 0 52.0 ± 0 151.0 ± 0 158.0 ± 0 105.0 ± 0 32.0 ± 0	211.8 ± 53.9 96.7 ± 63.2 53.2 ± 13.24 141.0 ± 55.0 143.6 ± 24.3 105.2 ± 34.6 62.8 ± 44.1			0.616 0.674 0.900 0.792 0.867 0.880 0.588
**Mediterranean Diet Adherence (Kid Med Index)**						
**Children’s Serum Lipid Levels**	**Low (<4)** **(*n* = 4)**	**Medium (4–7)** **(*n* = 20)**	**High (>7)** **(*n* = 7)**			***p-v***
Total Cholesterol (mg/dL) Triglycerides (mg/dL) HDL cholesterol (mg/dL) LDL cholesterol (mg/dL) ApoA (mg/dL) ApoB (mg/dL) LpA (mg/dl)	213.0 ± 9.8 45.2 ± 9.3 65.5 ± 20.8 138.5 ± 14.1 159 ± 19.4 86.2 ± 14.0 27.3 ± 19.4	212.6 ± 57.6 107.8 ± 65.0 50.9 ± 12.2 140.5 ± 58.3 142.9 ± 22.8 110.3 ± 36.7 65.9 ± 42.7	224.5 ± 48.7 94.1 ± 99.1 55.0 ± 12.9 156.2 ± 51.7 127.6 ± 32.5 96.6 ± 22.7 57.7 ± 53.0			0.872 0.285 0.163 0.786 0.142 0.367 0.275

* *pv* <0.05 (highlighted with bold) represents statistically significant differences among lipid values in different answers. Different letters in each line represents the difference among the answers (one Way Anova, Bonferoni test).
